# Divergent Innervation of the Olfactory Bulb by Distinct Raphe Nuclei

**DOI:** 10.1002/cne.23713

**Published:** 2015-01-14

**Authors:** Raphael Steinfeld, Jan T Herb, Rolf Sprengel, Andreas T Schaefer, Izumi Fukunaga

**Affiliations:** 1 Behavioural Neurophysiology, Max Planck Institute for Medical ResearchHeidelberg, 69120, Germany; 2 Champalimaud Centre for NeuroscienceLisbon, 1400–038, Portugal; 3 Institute for Anatomy and Cell Biology, University of HeidelbergHeidelberg, 69120, Germany; 4 Division of Neurophysiology, MRC National Institute for Medical ResearchLondon, NW7 1AA, UK; 5 Department of Molecular Neurobiology, Max Planck Institute for Medical ResearchHeidelberg, 69120, Germany; 6 Department of Neuroscience, Physiology and Pharmacology, University College LondonWC1E 6BT, UK

**Keywords:** quantitative image analysis, rAAV, RRIDs: AB_300798, AB_1142794, nlx_153890

## Abstract

The raphe nuclei provide serotonergic innervation widely in the brain, thought to mediate a variety of neuromodulatory effects. The mammalian olfactory bulb (OB) is a prominent recipient of serotonergic fibers, particularly in the glomerular layer (GL), where they are thought to gate incoming signals from the olfactory nerve. The dorsal raphe nucleus (DRN) and the median raphe nucleus (MRN) are known to densely innervate the OB. The majority of such projections are thought to terminate in the GL, but this has not been explicitly tested. We sought to investigate this using recombinant adeno-associated viruses (rAAV)-mediated expression of green fluorescent protein (GFP)-synaptophysin targeted specifically to neurons of the DRN or the MRN. With DRN injections, labeled fibers were found mostly in the granule cell layer (GCL), not the GL. Conversely, dense labeling in the GL was observed with MRN injections, suggesting that the source of GL innervation is the MRN, not the DRN, as previously thought. The two raphe nuclei thus give dual innervation within the OB, with distinct innervation patterns. J. Comp. Neurol. 523:805–813, 2015. © 2015 Wiley Periodicals, Inc.

Modulation of brain activities, that is, how a given circuit operates in seemingly different modes, has been observed in many brain regions and is likely to be a general property of brain operation across species (Marder, 2012; Bargmann and Marder, 2013; Linster and Fontanini, 2014). Such modulation is thought to involve coordinated changes in a variety of neurons orchestrated by a diffuse network of fibers containing neurotransmitters collectively known as neuromodulators. In contrast to the diffuse and widespread presence of such fibers, neurons containing such neurotransmitters are found in distinct and relatively small nuclei (Dahlstroem and Fuxe, 1964; Nieuwenhuys et al., 1988). The neurotransmitter 5-hydroxytryptamine (5-HT), also known as serotonin, is one of the most studied neuromodulators to date. Collections of serotonergic neurons found in the brain stem known as the raphe nuclei are the brain's major sources of serotonin (Dahlstroem and Fuxe, 1964; Steinbusch, 1981; Jacobs and Azmitia, 1992). What role serotonin plays is an active area of investigation and direct, experimental demonstrations of serotonergic neuromodulation are beginning to emerge on diverse fronts. To give examples, the activity of dorsal raphe nucleus (DRN) neurons and the corresponding serotonin levels in cortical and subcortical areas are correlated with wakefulness and non-REM sleep characterized by a desynchronized pattern in the EEG (Jacobs and Azmitia, 1992; Thakkar et al., 1998; Portas et al., 2000). Serotonin has also been shown to underlie state transitions from roaming to dwelling behaviors in *Caenorhabditis elegans* (Flavell et al., 2014).

The olfactory system, especially the olfactory bulb (OB), is a major recipient of serotonergic innervation (Steinbusch, 1981; McLean and Shipley, 1987; Datiche et al., 1995; Shipley and Ennis, 1996). The OB is also a layered structure, where interneurons present in distinct layers are involved in unique functions, such as signal transformation on different timescales (Fukunaga et al., 2014; Linster and Fontanini, 2014). Dense serotonergic innervation, particularly in the glomerular layer (GL), has been described (McLean and Shipley, 1987). As the first complex processing of olfactory signals occurs in glomeruli, neuromodulation here has a potentially major effect on olfactory functions. For example, activation of 5HT_2C_ receptors in the glomerular layer can excite local interneurons (Hardy et al., 2005; Petzold et al., 2009), in turn inhibiting the olfactory nerve terminals, indicating that serotonergic inputs in the GL can in effect gate incoming olfactory signals (Petzold et al., 2009). In vitro studies also demonstrate that excitatory neurons of the OB, including external tufted and mitral cells, can be activated directly by serotonin (Hardy et al., 2005; Liu et al., 2011; Schmidt and Strowbridge, 2014), outside of the GL (Schmidt and Strowbridge, 2014).

Retrograde tracing studies show that the DRN and the median raphe nucleus (MRN) both provide serotonergic inputs to the OB (McLean and Shipley, 1987). The DRN has also been linked, through direct electrical stimulation, to modulation of glomerular layer physiology (Petzold et al., 2009). The DRN and MRN are, however, known to be distinct, with mostly nonoverlapping projection patterns in other brain regions (Jacobs and Azmitia, 1992). It is therefore a prerequisite to establish their innervation pattern in the OB for studying how neuromodulation is implemented in the OB.

Earlier anatomical studies did not explicitly distinguish the two sources of ascending inputs to the OB. We sought to revisit this question by using recombinant adeno-associated viruses (rAAVs) for anterograde tracing. Specifically, we expressed enhanced green fluorescent protein (EGFP) fused to synaptophysin to augment visualization of presynaptic terminals (Nakata et al., 1998; Li and Murthy, 2001; Oh et al., 2014). We thus aimed to target distinct raphe nuclei with small injections to establish the precise termination patterns of ascending projections in the olfactory bulb, specifically with respect to the distinct layers.

## MATERIALS AND METHODS

Animal experiments were performed in compliance with the German animal welfare law. rAAV was prepared as previously described (During et al., 2003). A mixture of rAAVs was used for expression of EGFP-synaptophysin fusion protein under the control of the tTA-dependent promoter (Ptet; Zhu et al., 2007) and codon-improved tTA (itTA) under the synapsin or cytomegalovirus (CMV) promoter (Tang et al., 2009). The EGFP-synaptophysin fusion protein composed of EGFP (Zolotukhin et al., 1996) followed by a flexible linker region (AA positions 240–276) and synaptophysin (*Mus musculus*; GB accession number CAA65084; positions AA277–308), with the start codon Met removed. Note that the expression is not required to be conditional.

### Stereotactic injections

Mice (C56Bl/6N between 29 and 31 days of age, 15–16 g body mass) were anesthetized with ketamine/xyalzine (100 mg/kg and 20 mg/kg intraperitoneally; Inresa Arzneimittel, Freiburg, Germany) and placed in a stereotaxic frame (Kopf Instruments, Tujunga, CA). Lambda and bregma were leveled using an eLeVeLeR (Sigma Technology Systems, East Petersburg, PA) to 12° with bregma lower and a small craniotomy was made at the following coordinates (relative to the bregma): X = 0 mm; Y = –4.525 mm; Z = –2.230 mm for DRN and X = 0 mm, Y = –4.780 mm; Z = –3.350 mm for MRN; 7.9–18.4 nl of rAAV was dispensed through a capillary glass (tip diameter 19–25 μm) using a Nanoject II (Drummond Scientific, Broomall, PA) at 2.3 nl every 10 seconds.

### Histology

Solutions used for histology were in phosphate-buffered saline (PBS) that contained (in mM): NaCl (137), KCl (2.8), KH_2_PO_4_ (1.5), Na_2_HPO_4_ (8.1) with pH adjusted to 7.4. All chemicals listed were from Sigma-Aldrich (St. Louis, MO) or Merck (Darmstadt, Germany) unless otherwise stated. Fourteen days after the virus injection, animals were sacrificed and perfused transcardially with 4% formalin (pH = 8.9). The whole brain was dissected out, postfixed overnight in 4% formalin at room temperature, washed in PBS, embedded in gelatin (10%), and further fixed overnight in 4% formalin before sagittal sections 50–75 μm thick were cut using a vibratome (Microm HM 650 V; Sigmann Elektronik, Hüffenhardt, Germany).

Alternate sections were treated for Nissl staining with cresyl violet or immunohistochemistry (chicken anti-GFP and goat anti-serotonin AbCam, Cambridge MA; RRIDs: AB_300798 and AB_1142794, respectively; see Table1). For immunohistochemistry, the sections were incubated in blocking solution (2% bovine serum albumin [BSA], 2% cold fish gelatin, 1% Triton-X100) for 30 minutes, washed in PBS, and incubated with the primary antibodies (1:1,000, diluted to 0.04% blocking solution) for 24 hours. Subsequently, the sections were washed (3× 10 minutes) in PBS, incubated with the secondary antibody solution (diluted to 1:500 in 2 μg/ml DAPI (4′,6-diamidino-2-phenylindole), and 0.04% blocking solution for 12 hours. Secondary antibodies used for serotonin and EGFP stainings were antigoat conjugated to Cy3 and antichicken conjugated to Alexa Fluor 488 (both diluted to 1:500; Jackson ImmunoResearch Laboratories, West Grove, PA). The antibody against GFP is used widely and known to stain various reporter lines bearing EGFP transgene but not wildtype animals. Distribution patterns of antiserotonin signals in the brainstem, as well as the OB, closely match the patterns reported earlier (Steinbusch, 1981; Takeuchi et al., 1982; McLean and Shipley, 1987; Zhang et al., 2013).

**TABLE 1:** Primary Antibody Information

Antigen	Immunogen	Manufacturer/Cat. No.	Dilution
Serotonin	Serotonin conjugated to BSA with paraformaldehyde.	AbCam, Cambridge, MA (ab66047) RRID: AB_1142794	1:1,000
GFP	Recombinant full-length protein	AbCam, Cambridge, MA (ab13970) RRID: AB_300798	1:1,000

**TABLE 2:** Abbreviations Used in Figures

Abbreviation	Key
OB	Olfactory bulb
GL	Glomerular layer
EPL	External plexiform layer
GCL	Granule cell layer
DRN	Dorsal raphe nucleus
MRN	Median raphe nucleus
mlf	Medial longitudinal fascicle
CLI	Caudal linear nucleus of raphe
scp	Superior cerebellar peduncle
DTg	Dorsal tegmentum

### Image processing

Confocal fluorescence images were obtained with a Leica SP 5 (Leica Microsystems, Mannheim, Germany) with a ×63 glycerol immersion objective (HCX PL APO, NA 1.30, Leica). Laser wavelengths of 405, 458, or 561 nm were used for DAPI, Alexa Fluor 488, or Cy3 signals, respectively, and recorded separately. The pictures were stitched with Leica Confocal Software (v. 2.6.0.7266, Leica Microsystems, Mannheim, Germany). Nissl-stained sections were imaged with a brightfield microscope (Stemi SV11, Zeiss, Oberkochen, Germany). All images were analyzed in Fiji (Schindelin et al., 2012) and MatLab (MathWorks, Natick, MA; RRID: nlx_153890). Fluorescently labeled cells in the DRN and MRN were manually counted (repeated three times to account for manual error). Identified cells were marked independently in the red and green channels and colocalization was determined by the proximity of the labels, with a cutoff distance of 12 μm. Detected colocalization was robust to minor changes in the cutoff distance. Subdivisions of the raphe nucleus were manually delineated based on structures visible in the adjacent Nissl-stained sections and 5-HT immunoreactivity in the same section. Definitions of the MRN and the DRN were based on Jacobs and Azmitia (1992), corresponding to B8 and B5 (also known as the nucleus centralis superior) and B7 and B6 (Dahlstroem and Fuxe, 1964), respectively.

### Detection of labeled fibers in the OB

For each animal, a coronal section approximately at the middle level of anteroposterior axis of the OB was selected for analysis. The results are qualitatively the same at various anteroposterior levels (data not shown). Confocal fluorescent images taken at high resolution (×63) were tiled together to reconstruct the entire coronal section. Ten samples (120 × 120 μm each) were taken from each OB layer, based only on the structures visible in the DAPI channel in order not to be biased by the locations of labeled fibers. Samples covered all mediolateral and dorsoventral positions equally, where approximately one sample was placed randomly within each 36° sector. Different sampling coordinates were used for each animal, as the shape of the OB differed between animals, ensuring that samples placed in distinct OB layers did not sample other layers. Each sample was denoised ("despeckle" function) and the background subtracted ("subtract background" function with radius set to 30 μm) in Fiji. The mean and standard deviation of pixel values from all samples were calculated for each animal ("global mean and standard deviation" per animal). Fluorescent signal was defined to be pixel values above the global mean + 6 standard deviations. Our overall findings are not sensitive to the exact cutoff values. Signal density was defined as the number of detected pixels normalized by the total number of pixels analyzed, and calibrated by the image resolution (∼7 pixels/1 μm) to obtain the units reported (μm^2^/100 μm^2^). Colocalization measure of EGFP and serotonin signals was based on a method previously described (van Steensel et al., 1996). Briefly, Pearson's correlation coefficients were calculated between red and green pixel intensities over a range of image shift (dx) for each volume. A presence of colocalization results in a distinct peak at dx = 0 (van Steensel et al., 1996). This was compared against image sets where one channel was rotated by 90° (control rotated images).

## RESULTS

### Serotonergic fibers densely innervate the glomerular layer

**Figure 1:**
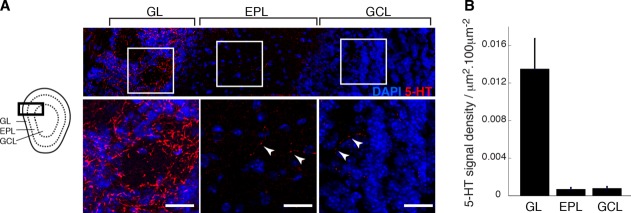
OB is densely innervated by serotonergic fibers. (A) Left: Illustration of a coronal section of the mouse main olfactory bulb showing the layered structure: GL = glomerular layer, EPL = external plexiform layer and GCL = granule cell layer. Top: Maximum projection showing 5-HT immunofluorescence (red) with respect to the OB layers. DAPI signal appears blue. Areas demarcated by white boxes are magnified below. White arrowheads indicate examples of 5-HT immunofluorescence in the EPL and GCL. (B) Summary quantification of serotonin immunofluorescence in the GL, EPL, and GCL (*n* = 7 animals). Scale bars = 20 μm.

Neurons in the OB are arranged in distinct layers (Fig. 1A) occupied by distinct interneuron classes (Shepherd, 1974). It is therefore important to resolve the serotonergic innervation pattern with respect to these layers. To this end, we carried out immunohistochemistry using an antibody against 5-HT. Intense labeling was observed in fibers located in the glomerular layer (0.014 ± 0.003 μm^2^/100 μm^2^, *n* = 7 animals; Fig. 1A,B). In addition, some fibers in the EPL and GCL were labeled, but were sparser (0.0007 ± 0.0002 μm^2^/100 μm^2^ and 0.0008 ± 0.0002 μm^2^/100 μm^2^ in EPL and GCL, respectively, *P* < 0.01, one-way analysis of variance [ANOVA] across all layers, *n* = 7 animals; Fig. 1A,B). No brightly labeled somata were visible, confirming previous reports that 5-HT is most densely located in the GL but its origin is purely extrinsic (Takeuchi et al., 1982; McLean and Shipley, 1987; Zhang et al., 2013).

### DRN projects to the OB but not to the glomerular layer

**Figure 2:**
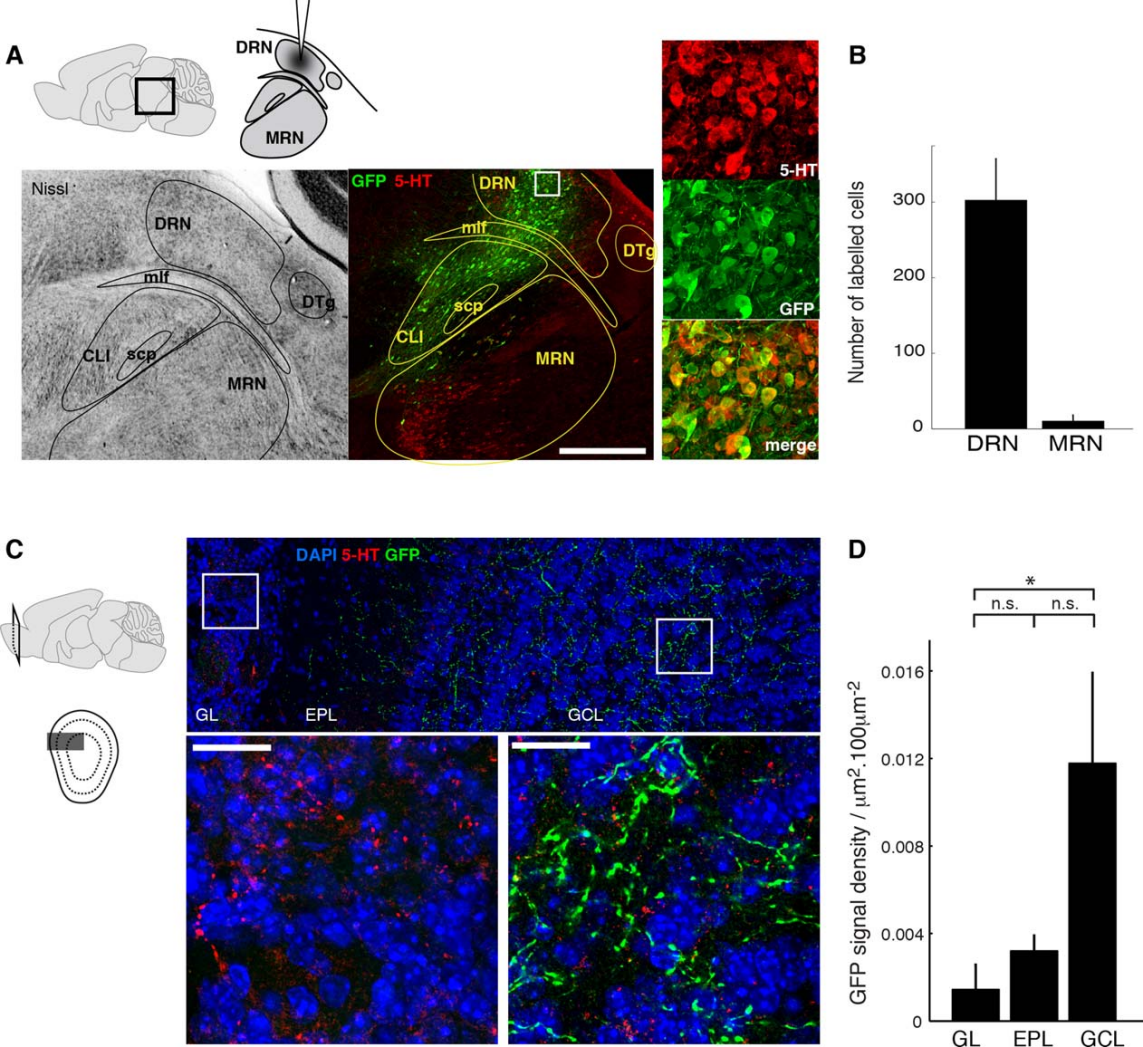
DRN projects preferentially to the GCL, not the GL. (A) Experimental configuration (top left): rAAV was injected into the DRN for expression of EGFP-synaptophysin for anterograde tracing. Bottom: Example of injection site (sagittal view); anatomical details visible in a Nissl-stained, adjacent section (left) were used to demarcate relevant structures in the fluorescent image (middle; maximum projection, green and red signals indicate EGFP and 5-HT immunofluorescence, respectively). Region demarcated in the white box is shown on the right. Dorsal raphe nucleus (DRN), mlf (medial longitudinal fascicle), CLI (caudal linear nucleus of raphe), superior cerebellar peduncle (scp), median raphe nucleus (MRN) and dorsal tegmentum (DTg). Magenta-green copies of the fluorescent images are available in Supplementary Fig. 1. (B) Summary quantification; numbers of infected cells counted in DRN or MRN. *n* = 4 animals. (C) Location of EGFP-labeled fibers within the OB following DRN injections. Left: Schematic showing the approximate location where the OB images below were taken. Top: Overview image showing the OB layers seen with DAPI (blue) overlaid with immunofluorescence for 5-HT (red) and EGFP (green; maximum projection depicted). Squares indicate the areas magnified below. Bottom: Example high-magnification image from the GL (left) shows dense 5-HT signal (red) but no EGFP signal while the example from GCL (right) shows dense EGFP labeling. Three color channels as above. See magenta-green copy in Supplementary Fig. 2. (D) Summary quantification showing EGFP signal densities in GL, EPL, and GCL for DRN injections. *P = 0.043, n.s. = not significant; *P* = 0.095 and 0.89 for EPL vs. GCL and GL vs. EPL, respectively; Tukey HSD post-hoc tests. Scale bars = 0.5 mm in A; 20 μm in C.

Previous reports have strongly linked the DRN to serotonergic modulation in the GL (McLean and Shipley, 1987; Petzold et al., 2009). To test whether the serotonergic fibers here originate in the DRN, we made small injections targeted to the DRN using the rAAVs for EGFP-synaptophysin expression (see Materials and Methods). Four out of five animals injected were found to have substantial infection in the dorsal raphe, confirmed by the location of 5-HT immunoreactivity and nearby structures visible in Nissl stains in the adjacent sections in each animal (Fig. 2A). An analysis of the injection site revealed that 303 ± 55 cells expressed EGFP in the planes treated for immunohistochemistry (Fig. 2A,B), where 43 ± 60 cells were additionally immunoreactive for 5-HT, indicating that significant numbers of DRN serotonergic neurons were infected. Importantly, the injections left MRN largely uninfected (11 ± 8 cells infected in MRN; Fig. 2A,B).

Following the DRN injections, numerous brightly labeled fibers were visible in the OB (Fig. 2C). The majority of the labeled fibers were located in the GCL (EGFP signal density in GCL = 0.012 ± 0.004 μm^2^/100 μm^2^; 70 ± 12% of signal in the OB). EGFP labeling was hardly present in the glomerular or the external plexiform layer (0.001 ± 0.001 μm^2^/100 μm^2^ and 0.003 ± 0.001 μm^2^/100 μm^2^ in GL and EPL, respectively, *P* = 0.04, one-way ANOVA across layers; Fig. 2D), even though immunostaining confirmed the ample presence of 5-HT-containing fibers in the GL for each animal.

### MRN is the source of serotonergic fibers in the GL

**Figure 3:**
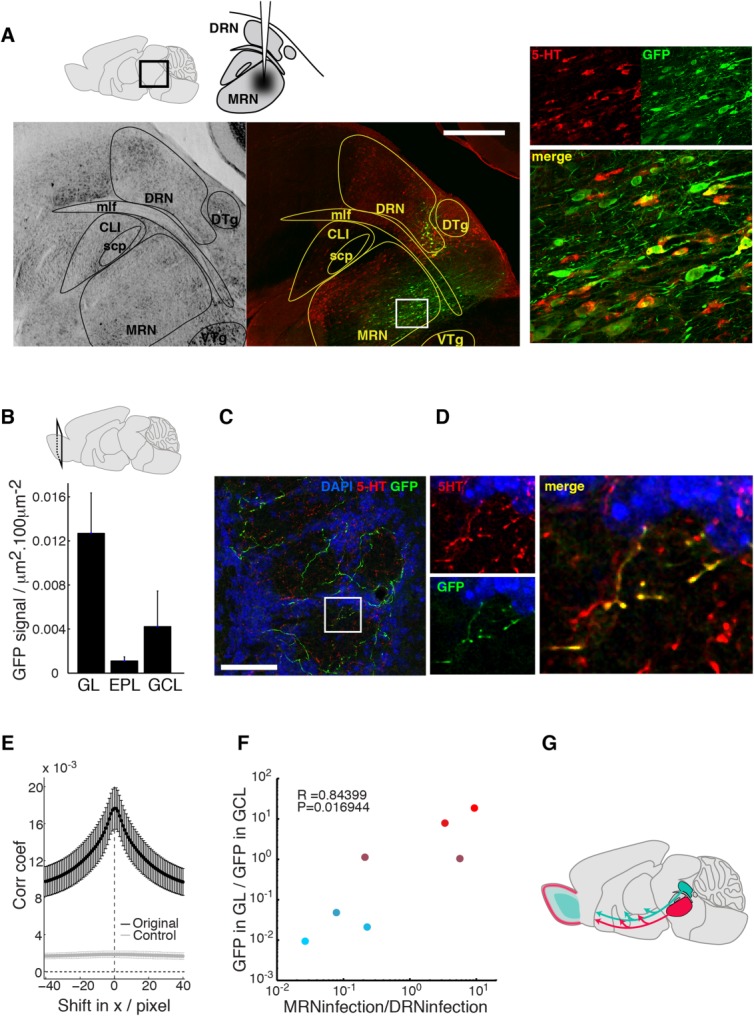
MRN is the likely source of serotonergic innervation in the GL. (A) Experimental configuration (top left): rAAV injections were targeted to the MRN. Example Nissl (bottom left) and fluorescent (middle) images from adjacent sections (maximum projection; red and green signals = 5-HT and EGFP immunofluorescence, respectively) showing infections successfully targeted to the MRN. Region demarcated by the white box is shown magnified (right). Magenta-green copy in Supplementary Fig. 3. (B) Summary quantification of EGFP signals in GL, EPL, and GCL following MRN injections (*n* = 3 animals). (C) Example from the glomerular layer of the OB; numerous EGFP-labeled fibers (green; maximum projection) in glomeruli are present. DAPI and 5-HT signals appear blue and red, respectively. (D) Magnified images from area demarcated on the left image. Some of the EGFP signals (left bottom, green) and 5-HT signals (left top, red) overlap in the merged image (right). Magenta-green copy in Supplementary Fig. 4. (E) Quantification of colocalization; Pearson's correlation coefficient for EGFP vs. serotonin signals plotted against image displacement in the x direction (dx). Control corresponds to image sets with one channel rotated by 90°. Mean ± SEM shown. Displacement in y direction gives equivalent result to dx, and thus not shown. (F) Summary plot for DRN and MRN injections; relative abundance of EGFP signals in the GL vs. GCL correlates strongly with relative infection levels in MRN vs. DRN, respectively. Color indicates relative abundance of GFP signals in distinct layers where [R G B] = [log(GFP_GL_/GFP_GCL_) 0.8*(1-log(GFP_GL_/GFP_GCL_)) (1- log(GFP_GL_/GFP_GCL_))]. (G) Schematic of the findings; DRN preferentially targets the GCL and EPL, while MRN projects to the GL. Scale bars = 0.5 mm in A; 50 μm in C.

The above results suggest that the DRN does not project to the GL, raising the question as to the source of the serotonergic innervation in the glomerular layer. The MRN is a potential source, as this nucleus also contains serotonergic neurons (Steinbusch, 1981; McLean and Shipley, 1987; Jacobs and Azmitia, 1992) and is reported to project to the OB (McLean and Shipley, 1987). In order to test this, we made small injections of rAAV-EGFP-synaptophysin viruses targeted to the MRN. Three out of six animals were confirmed to have sufficient numbers of MRN neurons infected (127 ± 26 infected neurons, Fig. 3A; 20 ± 6% immunoreactive for 5-HT). Some DRN neurons located immediately above the MRN were also infected (57 ± 39 cells, Fig. 3A).

Abundant labeling in the OB was observed following MRN injections. In particular, in stark contrast to the pattern observed with the DRN injections, the majority of the labeling was now in the GL (0.013 ± 0.004 μm^2^/100 μm^2^, 73.4 ± 12.4% of all label; Fig. 3B,C), and colocalized with 5-HT (peak correlation coefficient at 0.02 ± 0.40 pixel shift; *P* < 0.01 paired *t*-test for significant difference from the rotated image sets, *n* = 3; Fig. 3C–E). Furthermore, combining data from the two injection protocols revealed that the relative amount of labeled fibers in the GCL and GL corresponds tightly to the ratio of infected neurons in the DRN and MRN neurons (*r* = 0.84, *P* = 0.017, *n* = 7 animals; Fig. 3F), indicating that the termination pattern of afferents from the raphe is nucleus-specific, and only the MRN projects to the GL (Fig. 3G).

## DISCUSSION

Our localized injections in the two distinct raphe nuclei suggest that the DRN and the MRN have markedly different innervation patterns in the OB: the former projects mainly to the GCL, whereas the latter to the GL. Innervation in the EPL were relatively low in both DRN- and MRN-injected animals.

The lack of labeled fibers in the GL following DRN injections is particularly notable given the previous studies that linked the DRN directly to the serotonergic innervation and neuromodulation in the GL (McLean and Shipley, 1987; Petzold et al., 2009). One obvious consideration is whether the relevant neurons within the DRN have been targeted with our DRN injections. According to a previous retrograde tracing study, all four subdivisions of the DRN innervate the OB (MacLean and Shipley, 1987). Our injections covered various rostrocaudal extents, none of which resulted in appreciable labeling of fibers in the GL. The rich labeling in the GCL instead indicates that we targeted the portions of the DRN that project to the OB. It is unlikely that this labeling in the GCL came from unintended infection of other nuclei, such as the locus coeruleus, known to project to the GCL (McLean et al., 1989; Shipley and Ennis, 1996; Linster and Fontanini, 2014), as such nuclei are too far with our small injections to reach. The lack of attention so far given to the GCL innervation is likely due to the sparser serotonin labeling there, observed with antibodies against 5-HT or SERT alike (Takeuchi et al., 1982; McLean and Shipley, 1987; Zhang et al., 2013), in addition to not fully resolving different OB layers or distinct raphe divisions with previous wheatgerm agglutinin injections (McLean and Shipley, 1987). Similarly, it is possible that neuromodulatory effects in the GL that were previously observed through electrical stimulation of the DRN (Petzold et al., 2009) could be attributed to unintended recruitment of the MRN neurons, as they are located in close proximity to the DRN.

EGFP signals following the MRN injections clearly colocalized with serotonin at least partially in the GL (Fig. 3C–E), making it likely that the MRN is the source of serotonergic innervation here. The partial nature of colocalization observed may in part reflect the possible labeling of passing fibers with this method (see(Oh et al., 2014), as no additional methods, such as staining with FM dyes (Li and Murthy, 2001), were used to distinguish release sites. Collaterals from GL-targeting fibers (Suzuki et al., 2014) may partly explain some signals detected in the GCL following MRN injections, in addition to some contribution from DRN neurons that were also infected at the MRN coordinates. In turn, the dense EGFP labeling in the GCL following the DRN injections seems to outnumber the serotonergic labeling. In addition, no appreciable level of colocalization was detected, bringing into question whether the innervation in the GCL is serotonergic. McLean and Shipley (1987) reported that most anterogradely labeled DRN neurons were serotonergic; therefore, it is possible that the sparse 5-HT labeling here may relate instead to difficulty in detecting labeled fibers, particularly because fibers here are known to be thinner (McLean and Shipley, 1987). GCs themselves seem to express 5HT_1A_ receptors (Pompeiano et al., 1992), but so far in vitro experiments demonstrate a lack of direct GC activation by serotonin (Schmidt and Strowbridge, 2014). However, their postsynaptic partners need not be located in the immediate vicinity of release sites, as serotonin could propagate by volume transmission (Bunin and Wightman, 1999). Another possibility is that the DRN uses other neurotransmitters to innervate the GCL. Indeed, the DRN contains neurons that contain neurotransmitters other than serotonin (Jacobs and Azmitia, 1992), including GABA, dopamine, substance-P, and cholecycstokinin (Van Der Kooy et al., 1981; Fu et al., 2010) or glutamate colocalizing in serotonergic neurons (Fu et al., 2010), although some may only project locally (Van Der Kooy et al., 1981). Despite the ambiguity in the transmitter used, what is clear from our study is that the DRN is not the source of serotonergic innervation in the glomerular layer, as commonly assumed.

The nucleus-specific innervation pattern we observe here is reminiscent of largely nonoverlapping projection patterns from the raphe to other brain areas (Steinbusch, 1981; Jacobs and Azmitia, 1992). In addition, the GL and the GCL likely implement distinct functions within the OB, for example gating incoming signals in the GL (Petzold et al., 2009), or transforming signals on different timescales (Fukunaga et al., 2012, 2014; Linster and Fontanini, 2014). Whether the distinct projection patterns by the raphe translate to modulation of specific combination of areas or circuits within a region, and as a result distinct sets of functions, remains to be investigated. Our results indicate that at least in the OB, addressing layer selective neuromodulation may be possible by selective manipulation of distinct raphe nuclei.
