# Supplementary figures and images for: Divergent Innervation of the Olfactory Bulb by Distinct Raphe Nuclei
Source: J Comp Neurol. 2015 Jan 14;523(5):805–13. doi: 10.1002/cne.23713 (PMC4328392; doi:10.1002/cne.23713)

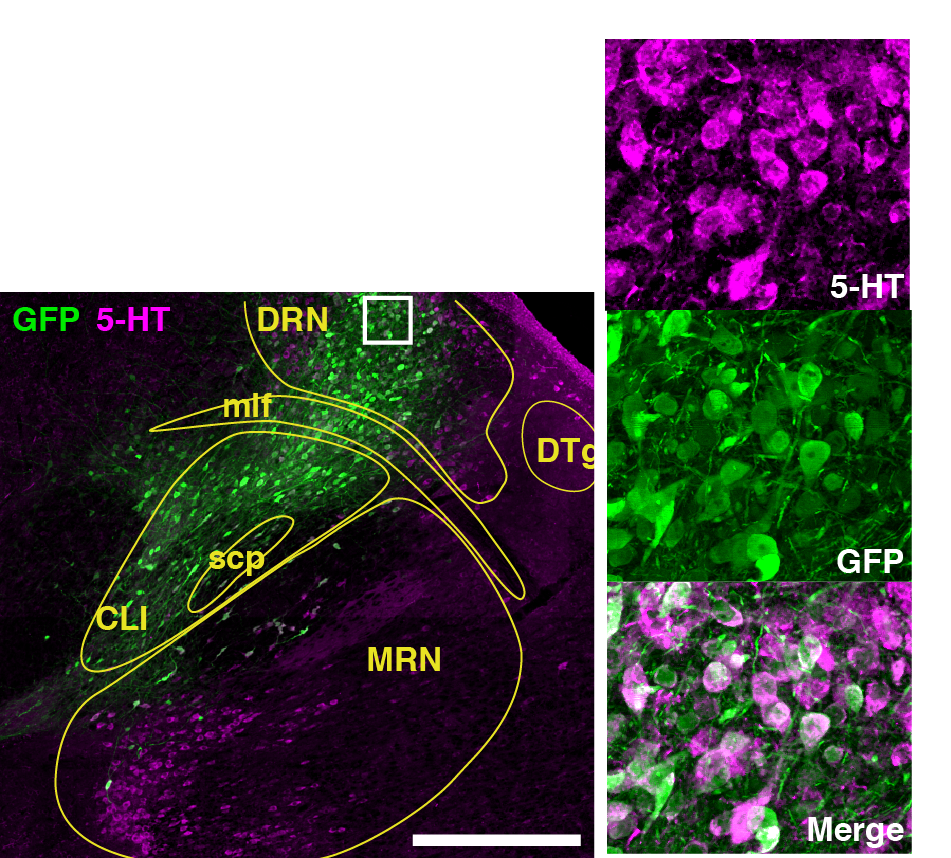

Supplement: Supplementary file 1 [file cne0523-0805-sd1.tif]

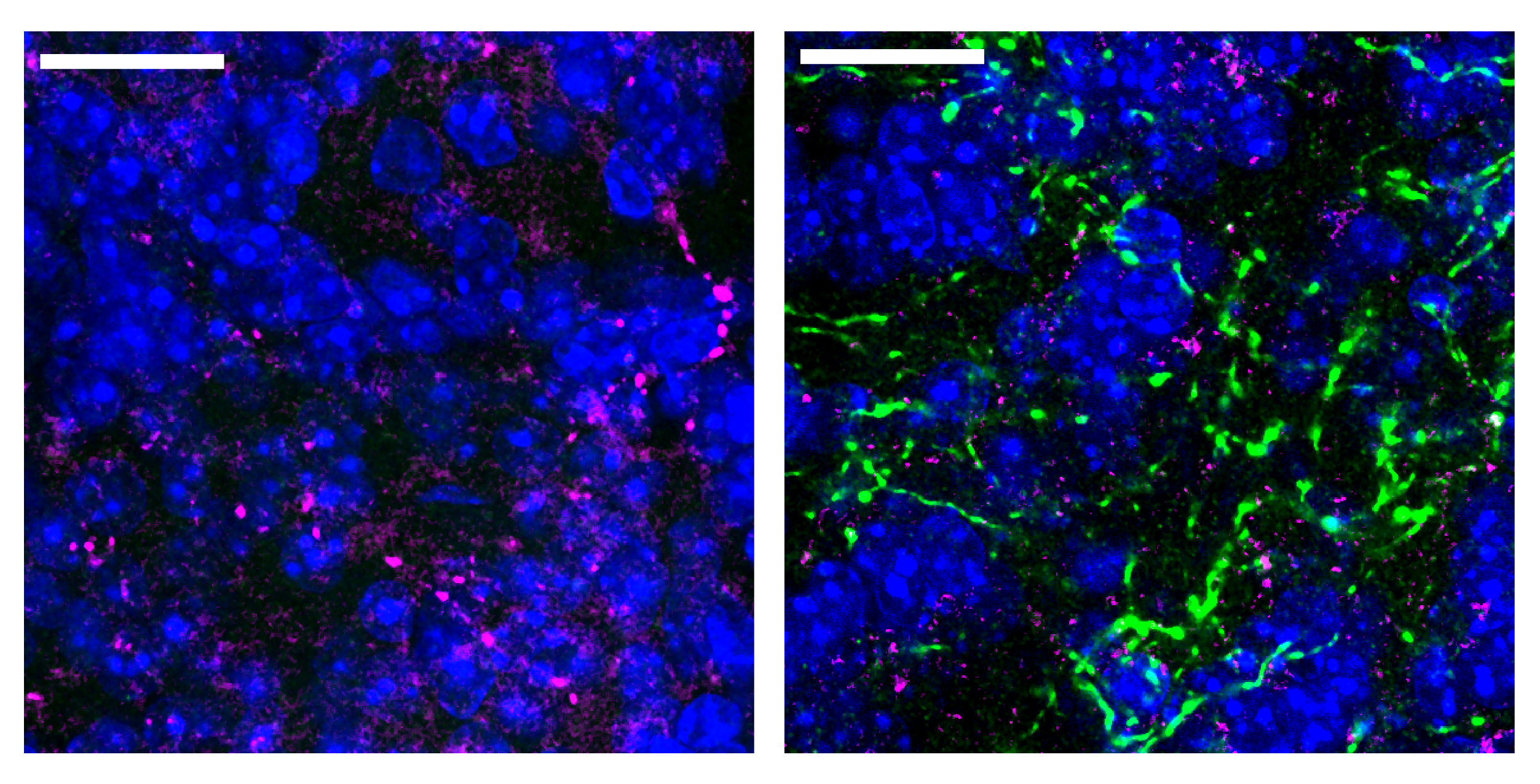

Supplement: Supplementary file 2 [file cne0523-0805-sd2.tif]

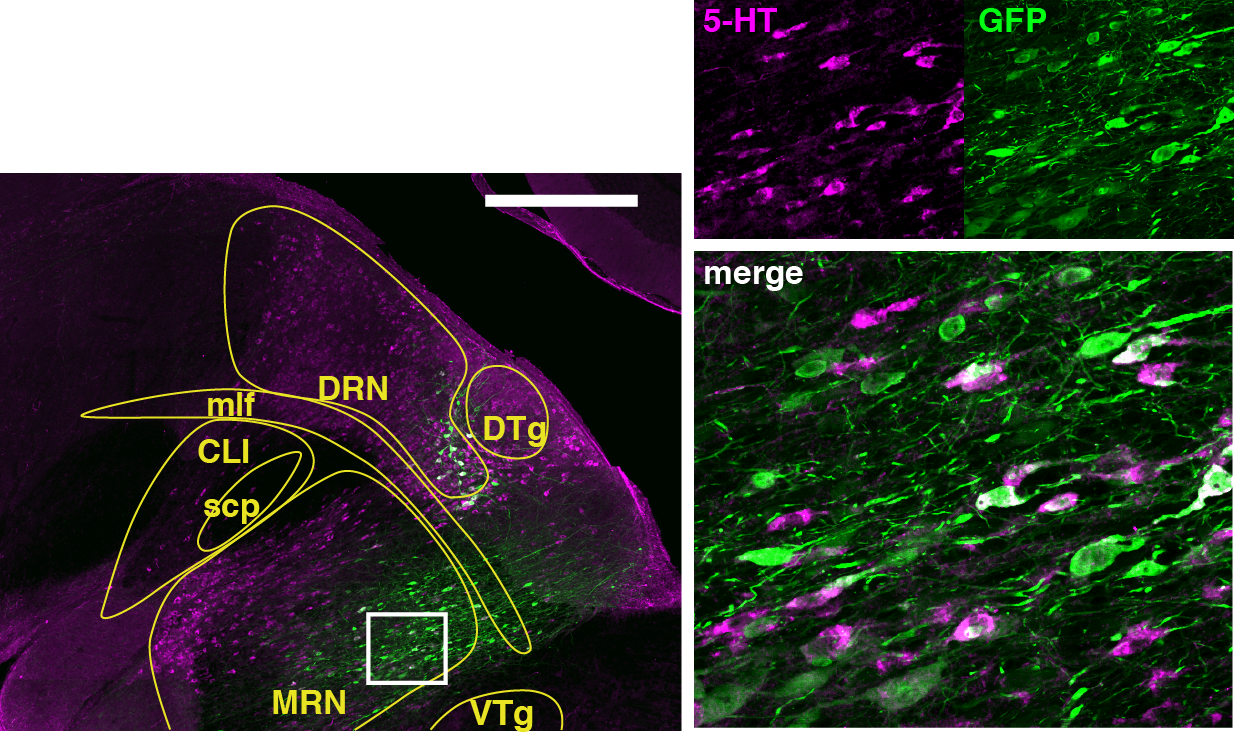

Supplement: Supplementary file 3 [file cne0523-0805-sd3.tif]

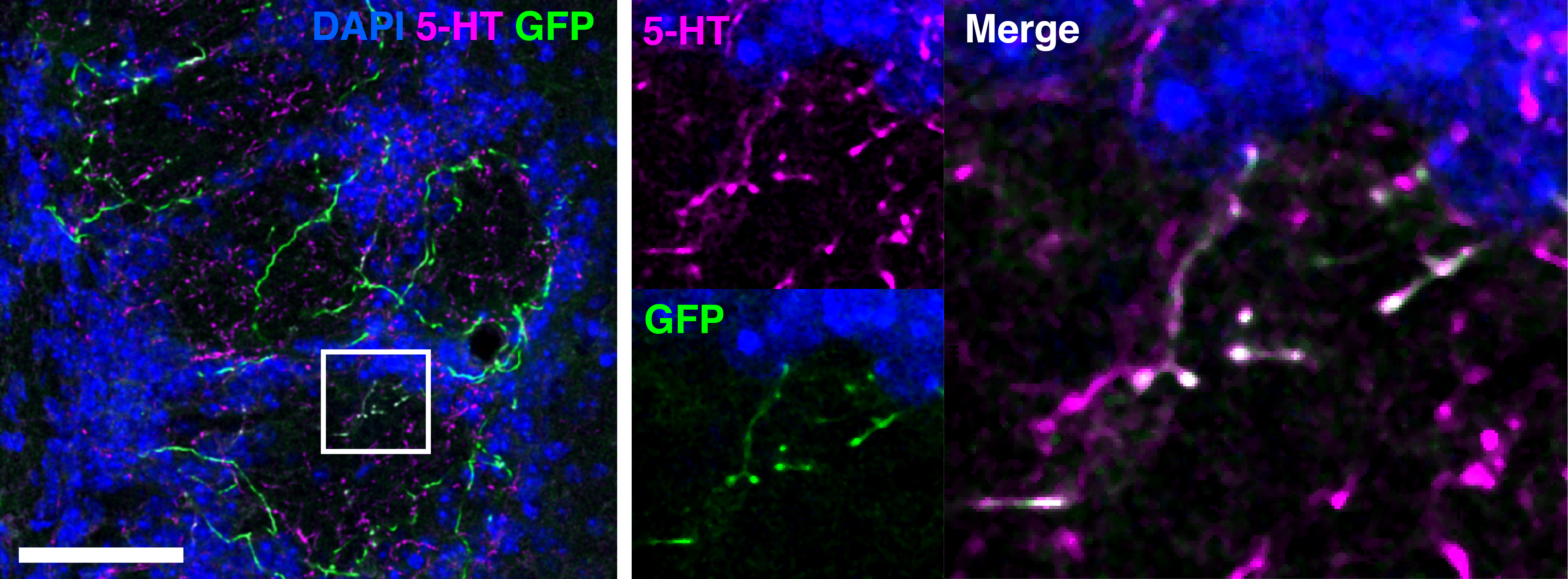

Supplement: Supplementary file 4 [file cne0523-0805-sd4.tif]
