# Supplementary material for: Divergent Innervation of the Olfactory Bulb by Distinct Raphe Nuclei
Source: J Comp Neurol. 2015 Jan 14;523(5):805–13. doi: 10.1002/cne.23713 (PMC4328392; doi:10.1002/cne.23713)
Supplement: Supplementary file 5 [file cne0523-0805-sd5.docx]

**Supplementary figure legends**

**Supplementary Figure 1**

Magenta-green copy of Figure 2A, with 5-HT and GFP signals indicated by magenta and green, respectively, at an example DRN injection site. Scale bar = 0.5 mm.

**Supplementary Figure 2**

Magenta-green copy of Figure 2C, showing 5-HT (magenta) and GFP (green) signals in the GL (left) and GCL (right) following a DRN injection. Scale bars = 20 μm

**Supplementary Figure 3**

Magenta-green copy of Figure 3A, showing 5-HT (magenta) and GFP (green) signals at an example MRN injection site. Scale bar = 0.5 mm.

**Supplementary Figure 4**

Magenta-green copy of Figure 3D, showing 5-HT (magenta) and GFP (green) signals in the GL following an MRN injection. Area demarcated by the white box in the overview (left) is zoomed in on the right. Scale bar = 50 μm.
